# An Integrated MCDM Model for Conveyor Equipment Evaluation and Selection in an FMC Based on a Fuzzy AHP and Fuzzy ARAS in the Presence of Vagueness

**DOI:** 10.1371/journal.pone.0153222

**Published:** 2016-04-12

**Authors:** Huu-Tho Nguyen, Siti Zawiah Md Dawal, Yusoff Nukman, Achmad P. Rifai, Hideki Aoyama

**Affiliations:** 1 Department of Mechanical Engineering, Faculty of Engineering, University of Malaya, Kuala Lumpur, Malaysia; 2 School of Integrated Design Engineering, Keio University, Tokyo, Japan; Southwest University, CHINA

## Abstract

The conveyor system plays a vital role in improving the performance of flexible manufacturing cells (FMCs). The conveyor selection problem involves the evaluation of a set of potential alternatives based on qualitative and quantitative criteria. This paper presents an integrated multi-criteria decision making (MCDM) model of a fuzzy AHP (analytic hierarchy process) and fuzzy ARAS (additive ratio assessment) for conveyor evaluation and selection. In this model, linguistic terms represented as triangular fuzzy numbers are used to quantify experts’ uncertain assessments of alternatives with respect to the criteria. The fuzzy set is then integrated into the AHP to determine the weights of the criteria. Finally, a fuzzy ARAS is used to calculate the weights of the alternatives. To demonstrate the effectiveness of the proposed model, a case study is performed of a practical example, and the results obtained demonstrate practical potential for the implementation of FMCs.

## Introduction

After the economic downturn in 2008, manufacturing companies began to implement advanced manufacturing technology (AMT) to improve the capacity of small-and medium-sized enterprises (SMEs) [1]. However, the adoption of AMT can require substantial investment, reconfiguration of the organizational structure and changes in the working culture [2]. ASEAN (the Association of Southeast Asian Nations) is developing its automotive industry based on the fundamentals of the global automotive industry. ASEAN industry receives investment from foreign companies for the production of components, modules and systems [3]. The adoption of systematic management and technological innovation is necessary for the survival of SMEs, especially for markets in developing countries [4].

The dramatic competition in the global manufacturing market for mechanical parts has caused manufacturers to improve their delivery times and set competitive prices for small and medium orders. Batch size is ever decreasing, and specific customer requirements for flexibility have caused flexible manufacturing cells and systems (FMC/FMS) to become a highly competitive manufacturing strategy in the late twentieth century [5, 6].

The material handling system (MHS) is a significant component of the FMC/FMS, and it plays a critical role in reducing manufacturing lead times, increasing the efficiency of material flow, improving facility utilization and increasing the productivity of manufacturing SMEs [7, 8]. Facility planning includes the design and arrangement of the MHS and equipment. Material handling costs are significant in manufacturing, contributing approximately 15–70% of the total manufacturing cost [9]. Several studies have shown that handling operations typically account for 30–40% of production costs [8, 10] or 30–75% of total costs, and improvements can decrease a company's operating costs by 15–30% [7]. In manufacturing SMEs, the use of FMCs is an essential strategy that can be implemented. Conveyors can be mobile or fixed devices used to transport materials between two fixed points continuously or intermittently within a stable workflow [11]. Conveyors are essential components of the MHS for loading and unloading and provide a good alternative in FMC/FMS [10]. Appropriate conveyor selection will reduce manpower requirements, electricity costs, production time, prices and delivery time, and thus increase profitability and productivity [12].

The decision-making process in conveyor selection for FMCs is complex and time-consuming because it involves qualitative and quantitative criteria. It is important to consider the selection in the design stage due to its significant influence on the outcome of the FMC/FMS layout. Moreover, conveyor selection for production operations is highly challenging because it depends on the availability of a suitable technology and configuration in the market [10]. In developing countries, most material handling systems are not compatible with the facility planning process used in manufacturing SMEs. Equipment such as machines, robots, and conveyors come from different suppliers in various countries. Consequently, manufacturing costs are increased and delivery time is slow [12], and integration is difficult. The importance of conveyor selection cannot be overstated. Today, with the wide range of technology available for conveyor handling, the selection of the most suitable conveyor alternative by a manufacturing SME from hundreds of types and suppliers is not a trivial task and requires a very complicated decision [7, 11, 13].

To choose the appropriate device, most engineers and managers base their decisions on textbooks, handbooks, manuals and personal experience in their professional fields, or on the advice provided by suppliers and experts in material-handling engineering [11, 12]. However, engineers are usually faced with the challenge of choosing the right equipment without the necessary experience or familiarity. Consultation services are very expensive and are not suitable for SMEs. The advice from suppliers is free, but suppliers want to persuade customers to buy their products, and their advice thus has low reliability for conveyor selection. In contrast, analytical models based on multi-criteria analysis have rarely been used for equipment evaluation and selection [11].

In this study, an MCDM model is developed for evaluating conveyors based on fuzzy AHP and fuzzy ARAS. In particular, several factors related to ergonomics and restructurability are identified and their role in the decision-making process is assessed.

## Survey of Related Work

MCDM is the most commonly used decision-making method in government, science, engineering, business, and management. It is built on the assumption of a complex world, and can improve the quality of decisions by making the decision more clear, reasonable, and effective [14]. Multi-criteria decision analysis has rarely been used for conveyor evaluation and selection. Interest in the development of a framework to solve this problem has attracted many researchers to help engineers and managers make quick decisions. The decision-making process for conveyor equipment selection involves multiple factors with both qualitative and quantitative attributes. For the assessment and selection of a multi-criteria highly hierarchical structure with a small number of alternatives, the MCDM method has proven to be very effective in narrowing the solutions to a few potential alternatives using experts' judgments [15, 16]. For more detail on MCDM, see Mardani, Jusoh and Zavadskas [17], who report on research performed during the two decades from 1994–2014.

Several studies have been presented in this field in recent years. For example, Hadi-Vencheh and Mohamadghasemi [12] developed a novel hybrid MCDM model using FWA (fuzzy weighted averages), fuzzy VIKOR (Vlsekriterijumska Optimizacija I KOmpromisno Resenje) and fuzzy TOPSIS (Technique for Order of Preference by Similarity to an Ideal Solution) for the selection of material handling equipment. A case study of conveyor selection (comparing pneumatic, chute, roller, and flat-belt conveyors) was performed to validate the proposed model. Eleven experts evaluated the criteria weights. The alternatives were ranked using fuzzy VIKOR, and the results were compared with results from fuzzy TOPSIS.

Anand *et al*. [10] stated that the selection of a materials handling system (MHS) in the design of an FMS is a complicated decision-making process due to the dependence of the process on multiple factors. They proposed the use of the analytic network process (ANP), which is capable of taking into account both the inner dependence and outer dependence among 35 or more factors in decision making. However, the ANP approach has a high computational complexity when the number of criteria considered is large, and it is thus not suitable for engineers and managers who need to make quick decisions to solve real problems on the shop floor. Onut *et al*. [8] extended the approach to evaluate the alternatives of an industrial truck, a conveyor, a rail system crane, an AGV, and a fixed crane based on 5 criteria including material, movement, method, cost, and area constraints using fuzzy ANP and fuzzy TOPSIS. Additionally, Tuzkaya *et al*. [18] proposed an integrated approach using fuzzy ANP and fuzzy PROMETHEE for MHE selection. Their case study of MHE selection in a manufacturing company in Istanbul, Turkey was used to evaluate and select the most suitable industrial trucks based on four criteria: operation, economics, environment, and strategy. Lashgari *et al*. [19] used the combination of fuzzy AHP, fuzzy ANP and fuzzy TOPSIS for MHE selection (a hydraulic shovel, cable shovel, dragline, wheel loader, and backhoe loader) in a case study of the Gole Gohar surface mine.

Mousavi *et al*. [20] presented a novel fuzzy grey MCDM for the evaluation and selection of MHE in an uncertain environment of a textile manufacturing company. They developed an MCDM method based on the combination of compromise solutions and grey relational models for decision making in real-life situations.

Kulak [7] developed a decision support system (DSS) for fuzzy multi-attribute material handling equipment selection for the cases of both complete and incomplete information. The DSS compromised three components: (1) a database of equipment types and properties, (2) a rule-based system for determining the most suitable MHE type, and (3) an MCDM based on the information axiom of axiomatic design principles to select the best alternatives for four conveyors among the same types. Fonseca *et al*. [11] developed a prototype expert system for selecting industrial conveyors with the aim of providing solutions for MHE along with a list of potential vendors. Mohsen and Hassan [21] developed a framework to support practitioners, managers and expert systems developers in selecting MHE in manufacturing and logistics facilities. Chan *et al*. [22] developed an intelligent decision support system for MHE selection using three components: (1) a database of potential equipment with significant properties, (2) a knowledge-based system to support MHE selection, and (3) an AHP method to select the most suitable equipment type. A numerical example of the best commercial AGV selection was performed to validate the developed approach. Additionally, Rao [23] applied many different methods (e.g., SAW-simple additive weighting, the WPM-weighted product method, AHP, a GTMA-graph theory and matrix approach, TOPSIS, and modified TOPSIS) to decision making in conveyor selection that had been suggested in previous work by Kulak [7]. Karande and Chakraborty [24] applied the weighted utility additive (WUTA) approach for MHE selection. A case study of conveyor selection presented by Kulak [7] was used to validate the proposed approach. The results from WUTA were compared with the existing methods of FUMAHES, GTMA, VIKOR, PROMETHEE and ELECTRE.

Yazdani-Chamzini [25] presented an integrated approach using fuzzy AHP and fuzzy TOPSIS for group MCDM in selecting the most appropriate MHE in an open pit mine project based on the three main criteria and fifteen sub-criteria. Additionally, Aghajani Bazzazi *et al*. [26] developed a good compromise solution using VIKOR for MCDM in complex systems. The combination of AHP and the entropy method was used to determine the weightage of the attributes. To validate their proposed model, a case study was used of surface mine equipment selection from the three alternatives of a shovel truck, loader truck, and belt conveyor. Finally, providing further information on MHE selection, Saputro *et al*. [15] present a review of issues related to MHE.

The additive ratio assessment (ARAS) approach is a new MCDM methodology developed in 2010 by Zavadskas and Turskis [14]. The ARAS approach uses simple relative comparisons to help decision makers understand the phenomena of the complex world. In their method, a utility function value determines whether the complicated relative efficiency of a potential alternative is directly proportional to the relative efficiency of the values and priority weights of the main attributes. The ARAS MCDM method and a variation [27] have been successfully applied in many fields such as construction [28–31], built environments [32–34], energy technologies [35], mechanical material selection [36], personnel selection [37, 38], waste dump site selection [39], heritage value [40], green supplier selection using fuzzy AHP-ARAS [41], and logistic-center location selection [42].

This literature review demonstrates that the MCDM approach has the potential to solve the MHE selection problem in uncertain environments. Many MCDM approaches have been proposed, such as AHP/ANP, TOPSIS, VIKOR, SAW and PROMETHEE. In particular, AHP is commonly used to determine the weights of factors, attributes, and criteria [43, 44]. These methods, however, cannot capture factors related to the imprecise and vague information in an uncertain manufacturing environment. Fuzzy sets can use linguistic items to convert human judgment into fuzzy numbers for evaluation. Moreover, other MCDM methods have been used in other applications that were not mentioned in the survey of related work. For example, the AHP has been widely used in MCDM for MHE selection in recent years. It is a theory of measurement based on pairwise comparisons and relies on experts' judgments to assign priority or weight scales. These scales are based on absolute human judgments of how important one component is versus another with respect to a desired criterion [45]. However, a significant limitation of the AHP and classical MCDM approaches is their difficulty in capturing uncertain information because experts’ judgments involving preferences are usually vague and imprecise. Therefore, the judgments cannot be evaluated using an exact numerical value, and priorities or weights for the attributes are not determined accurately. In an uncertain environment, crisp data cannot be used to handle practical situations. The use of linguistic items (e.g., low, medium, or high) as the natural representation for judgments in lieu of crisp values has been proven effective in the MCDM process. These properties can be incorporated into fuzzy sets by capturing the judgments of decision makers because fuzzy logic is able to convert their judgments into fuzzy numbers using linguistic variables [46]. Therefore, fuzzy logic plays a critical role in the decision-making process, and it is integrated into the AHP to provide more accurate decisions. However, the AHP requires many pairwise comparisons when the number of criteria considered is increased. Consequently, the use of fuzzy ARAS as a continuation of fuzzy AHP reduces the number of pairwise comparisons and minimizes the need to collect experts’ judgments.

To our best knowledge, there are no papers currently available in the literature on FMC management, fuzzy AHP or fuzzy ARAS that address conveyor selection in the design of FMC. Moreover, very few articles in the MCDM literature address the conveyor selection problem in manufacturing SMEs. Therefore, an MCDM framework that integrates fuzzy AHP and fuzzy ARAS for conveyor evaluation and selection is needed in the design of FMC.

## The Proposed Framework for Conveyor Evaluation and Selection

**Fig 1:**
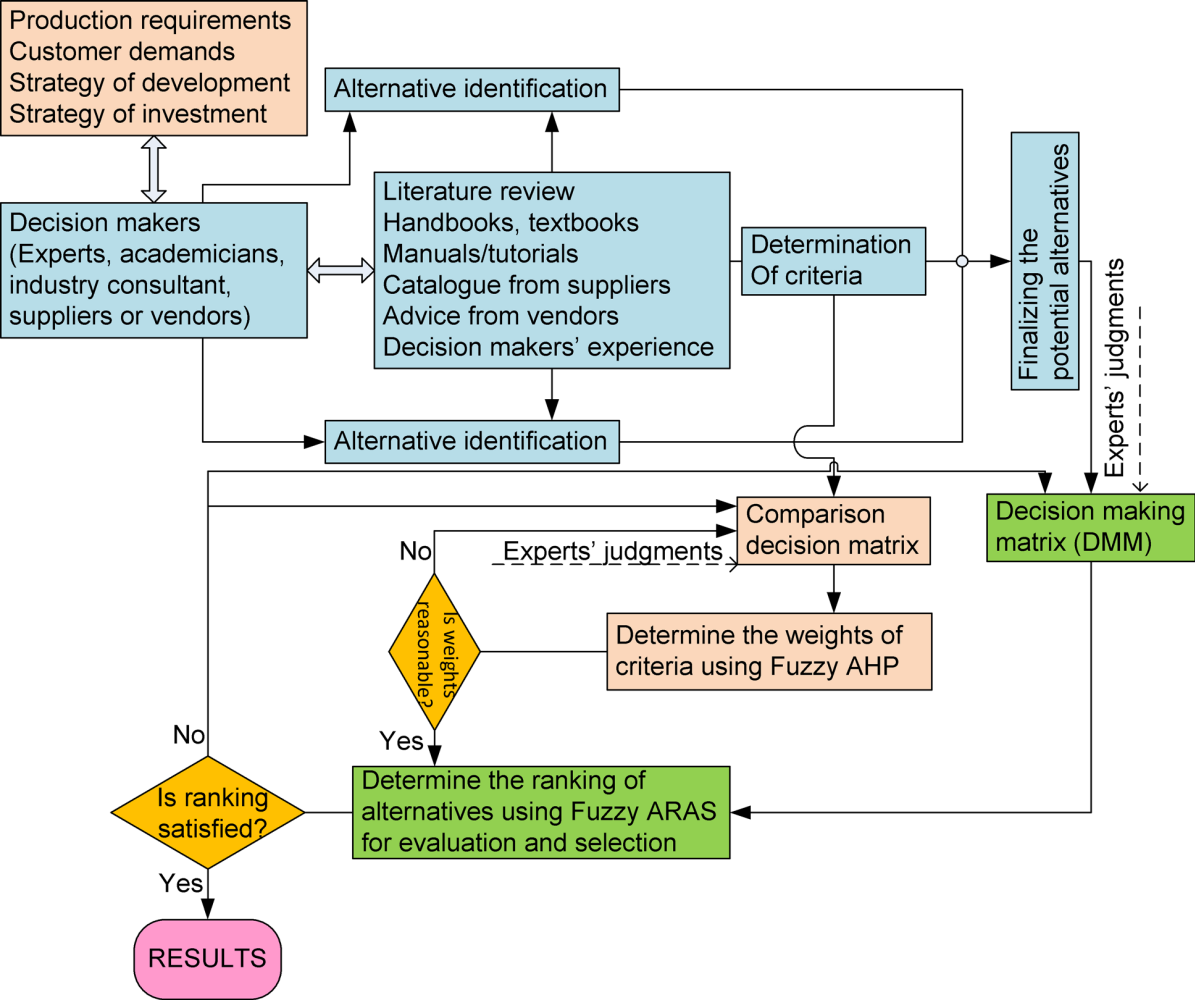
The proposed framework for the decision-making process.

The proposed framework consists of three parts, as shown in Fig 1. The first part is the determination of potential alternatives and the criteria that describe the alternatives based on input from decision makers. In particular, in addition to production requirements, customer demands, and development and investment strategies, the important criteria and potential alternatives are determined by the decision makers (such as experts, academicians, and industry consultants—in this paper, information about the decision makers is presented in Table 1, which is adapted from [47]) based on literature reviews, handbooks, textbooks, catalogues, advice from suppliers, and their own experience. The criteria and sub-criteria are selected based on the evaluation of a simple survey. The criteria and potential alternatives for the decision-making process are then finalized. Moreover, the hierarchical structure of the criteria is built to support the decision makers in the process of multi-criteria evaluation. Additionally, the weights for the criteria are calculated using fuzzy AHP. To do this, pairwise comparison matrices between criteria are collected based on the experts’ judgments. Fuzzy sets are used to convert the linguistic terms of the judgments into fuzzy numbers, which are quantitative. Finally, the ranking of alternatives is derived using the fuzzy ARAS approach. In this stage, the decision-making matrix is established by collecting the experts’ judgments evaluating each alternative for each criterion. If the ranking is not satisfied by the results from the decision makers, then the pairwise comparison matrices and the decision-making matrix must be reconsidered to check the consistency of the proposed model. The procedure is then repeated until the complete decision is satisfactory.

**Table 1:** Detailed information about the decision makers.

	Gender	Age	Education level	Experience (years)	Job title	Job responsibility
Decision maker 1 (DM1)	Female	55–60	Associate professor of mechanical engineering	>30	Expert in the field of material handling systems engineering, construction machinery and equipment.	Consultant in the design of material handling systems (e.g., elevators, conveyors, and cranes) and factory automation.
Decision maker 2 (DM2)	Female	45–50	Associate professor of manufacturing systems and ergonomics	>20	Expert in the design of manufacturing systems and ergonomics.	Work related to the evaluation of engineering projects in manufacturing systems and ergonomics; design of FMS/FMC.
Decision maker 3 (DM3)	Male	45–50	Associate professor of manufacturing processes	>20	Modern manufacturing processes and automation of manufacturing systems.	Supervision of the machining process; design and simulation of FMS/FMC.
Decision maker 4 (DM4)	Male	30–35	Master of construction machinery and material handling engineering	>7	Design of material handling manufacturing systems.	Evaluating multi-criteria projects in MHS and the design of mechanical conveyor systems.

**Fig 2:**
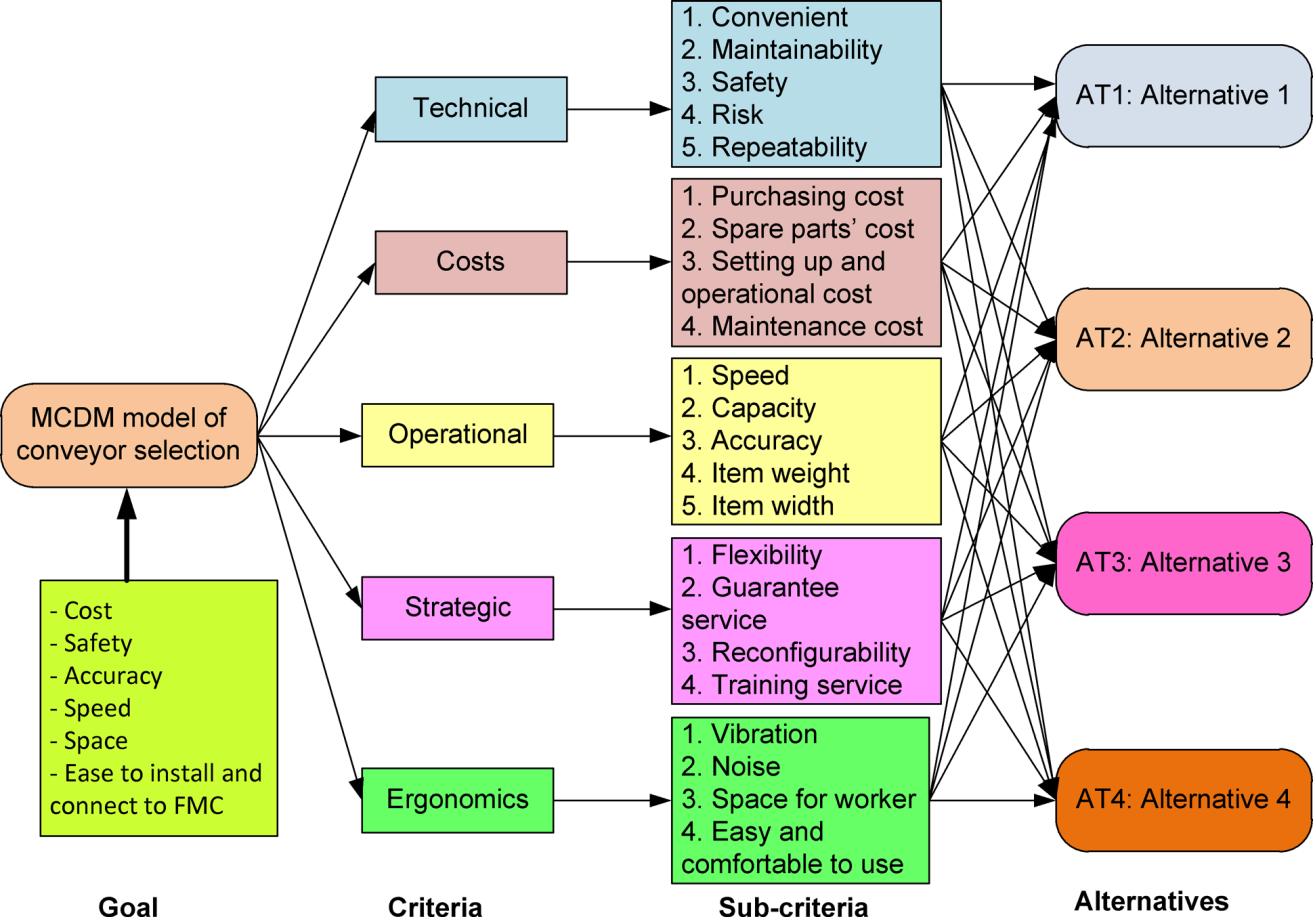
The hierarchical structure of the MCDM for conveyor evaluation and selection.

The hierarchical structure of the MCDM model comprises four levels as shown in Fig 2: (1) the production goal is a desired measurement of performance; (2) the five main criteria considered at this level are technical, cost, operational, strategic and ergonomic factors; (3) the 22 sub-criteria included in the model for evaluation are convenience, maintainability, safety, risk, repeatability, purchasing cost, spare parts cost, set-up and operational costs, maintenance cost, speed, capacity, accuracy, item weight, item width, flexibility, service guarantee, reconfigurability, training service, vibration, noise, space for the worker, and easy and comfortable use; and (4) the level of each potential alternative is evaluated based on all the criteria. All the criteria and sub-criteria were extracted selectively, based on the experts' opinions, from an ergonomic study [48] and an MHE selection study [12]. They were selected carefully and validated by an experienced industrial engineer. The meanings of the criteria used for conveyor evaluation and selection are explained in previous work by Rossi *et al*. [48]. We also added some novel ergonomic criteria and several sub-criteria, such as reconfigurability, which is the ability to rearrange the conveyor layout. Additionally, the hierarchical structure of the MCDM model was modified to be suitable for the conveyor selection process for FMC design in manufacturing SMEs.

## Research Methodology

AHP has the advantage of allowing the formation of a hierarchical structure of the criteria to support the decision-makers in focusing on the significant criteria when to determine its weights. Besides, the AHP method is simple and easy to use to assess the multi-criteria problem based on the scaling factors which establish the pairwise comparison matrices for different alternatives. However, this method has been criticized because of some reason in resolving the uncertainty and vagueness while determining the weights of criteria based on the experts’ judgments. Fuzzy sets theory is not capable of measuring the consistency of experts’ judgments and difficult to quantify the weights of the criteria in the decision hierarchy. But fuzzy set especially likely to address the qualitative and linguistic data, and provides a numerical representation method through linguistic variables to describe the experts’ judgments. Triangular fuzzy number (TFN) is commonly used in the decision-making process because of its simplicity and ease to understand and apply in the practice. Therefore, this paper integrates fuzzy sets and AHP to achieve the full advantage in the decision-making. Fuzzy AHP is a useful method, containing many outstanding advantages in addressing uncertainty and providing a fuzzy mathematical way to describe quantitative and qualitative data [49]. The combination of the most powerful features between fuzzy logic and AHP made fuzzy AHP require less computing power in making decision quickly. Therefore, it is potential for MCDM in the fuzzy environment, which was found to have sustainable applications in recent years [50]. The fuzzy AHP requires the large number of experts’ judgment needed to collect. So it is integrated with fuzzy ARAS to decrease the pairwise comparison matrices in determining the ranking of alternatives.

### Fuzzy sets and fuzzy numbers

Fuzzy set theory was introduced in 1965 by Zadeh [51] to handle uncertainty due to imprecise or vague information. A fuzzy set *A* = {(*x*, *μ*_*A*_(*x*)) | *x* ∈ *X*} is a set of ordered pairs, where X is a subset of the real numbers *R* and *μ*_*A*_(*x*) is a membership function that assigns to each objective x a score ranging from zero to one [52]. Fuzzy set theory is integrated into the pairwise comparison matrices of the AHP. Triangular fuzzy numbers (TFNs) are most commonly used to describe practical experts’ judgments and are represented as $A(\underline{a},a,\bar{a})$. The parameters ($\underline{a},a,\bar{a}$) are the smallest, intermediate (i.e., the most promising), and largest values used in defining the uncertain judgments. The fuzzy numbers used to evaluate a process in this study are described in Table 2. The general membership function of a TFN is determined as follows.

$$\mu \left(x/A\right)=\{\begin{array}{l} 0,\;x<\underline{a}\\ (x-\underline{a})/(a-\underline{a}),\;\underline{a}\leq x\leq a\\ (\bar{a}-x)/(\bar{a}-a),\;a\leq x\leq \bar{a}\\ 0,\;x>\bar{a} \end{array}$$ (1)

**Table 2:** Linguistic scale for importance.

Linguistic scale for importance	Triangular fuzzy scale
Just equal (JE)	(1,1,1)
Equally important (EI)	(1/2,1,3/2)
Weakly more important (WMI)	(1,3/2,2)
Strongly more important (SMI)	(3/2,2,5/2)
Very strongly more important (VSMI)	(2,5/2,3)
Absolutely more important (AMI)	(5/2,3,7/2)

For multi-criteria decision making, let *X* = {*x*_1_, *x*_2_,…, *x*_*n*_} be a set of criteria and *g* = {*g*_1_, *g*_2_,…, g_*n*_} be a set of alternatives; each alternative is identified over a set of criteria. Each criterion is then selected, and an extent analysis is employed for each alternative g_i_. Consequently, the value m of the extent analysis for each criterion is as follows:

$A_{gi}^{1},A_{gi}^{2},\ldots ,A_{gi}^{m}$, *i* = 1,2,…,*n*, where $A_{gi}^{j}(j=1,2,\ldots ,m)$ are the TFNs.

### Fuzzy AHP method

Let $A={\left(\tilde{a}\right)}_{n\times m}$ be a fuzzy pairwise comparison matrix, where $\tilde{a}=({\underline{a}}_{ij},a_{ij},{\bar{a}}_{ij})$. The procedure for the fuzzy AHP algorithm was first suggested by Chang [53, 54] and has been successfully applied in many fields. Several recent studies have used this procedure to determine criteria weights, such as Chen *et al*. [52], Prakash and Barua [55], Nguyen *et al*. [56, 57], Avikal *et al*. [58], Taylan *et al*. [59], Yi and Wang [60], Zhang and Deng et al. [61] and Bulut *et al*. [62]. For more details, Demirel et al. [63] also described the applications of fuzzy AHP and its variations. The steps of Chang's method [53] can be summarized as follows.

**Step 1**: The fuzzy synthetic extent value for the i^th^ criterion is determined from

$$S_{i}=\sum_{j=1}^{m}A_{gi}^{j}⊗{\left[\sum_{i=1}^{n}\sum_{j=1}^{m}A_{gi}^{j}\right]}^{-1}$$ (2)

where the value of $\sum_{j=1}^{m}A_{gi}^{j}$ is the fuzzy aggregation of m TFNs, which are the extent analysis values for a particular matrix.

$$\sum_{j=1}^{m}A_{gi}^{j}=\left(\sum_{j=1}^{m}{\underline{a}}_{j},\sum_{j=1}^{m}a_{j},\sum_{j=1}^{m}{\bar{a}}_{j}\right)$$ (3)

For the determination of the reciprocal value ${\left[\sum_{i=1}^{n}\sum_{j=1}^{m}A_{gi}^{j}\right]}^{-1}$, the fuzzy aggregation value of $A_{gi}^{j}(j=1,2,\ldots ,m)$ is calculated as follows.

$$\left[\sum_{i=1}^{n}\sum_{j=1}^{m}A_{gi}^{j}\right]=\left(\sum_{i=1}^{n}{\underline{a}}_{i},\sum_{i=1}^{n}a_{i},\sum_{i=1}^{n}{\bar{a}}_{i}\right)$$ (4)

The reciprocal value of the vector in the above equation is determined from

$${\left[\sum_{i=1}^{n}\sum_{j=1}^{m}A_{gi}^{j}\right]}^{-1}=\left(1/\sum_{i=1}^{n}{\bar{a}}_{i},1/\sum_{i=1}^{n}a_{i},1/\sum_{i=1}^{n}{\underline{a}}_{i}\right)$$ (5)

**Step 2**: The degree of possibility of $A_{2}=\left({\underline{a}}_{2},a_{2},{\bar{a}}_{2}\right)\geq A_{1}=\left({\underline{a}}_{1},a_{1},{\bar{a}}_{1}\right)$ is defined as

$$V\left(A_{2}\geq A_{1}\right)=\text{height}\left(A_{1}\cap A_{2}\right)=\{\begin{array}{l} 1,\;a_{2}\geq a_{1}\\ 0,\;{\underline{a}}_{1}\leq {\bar{a}}_{2}\\ \frac{{\underline{a}}_{1}-{\bar{a}}_{2}}{\left(a_{2}-{\bar{a}}_{2})-(a_{1}-{\underline{a}}_{1}\right)},\;\text{otherwise} \end{array}$$ (6)

**Fig 3:**
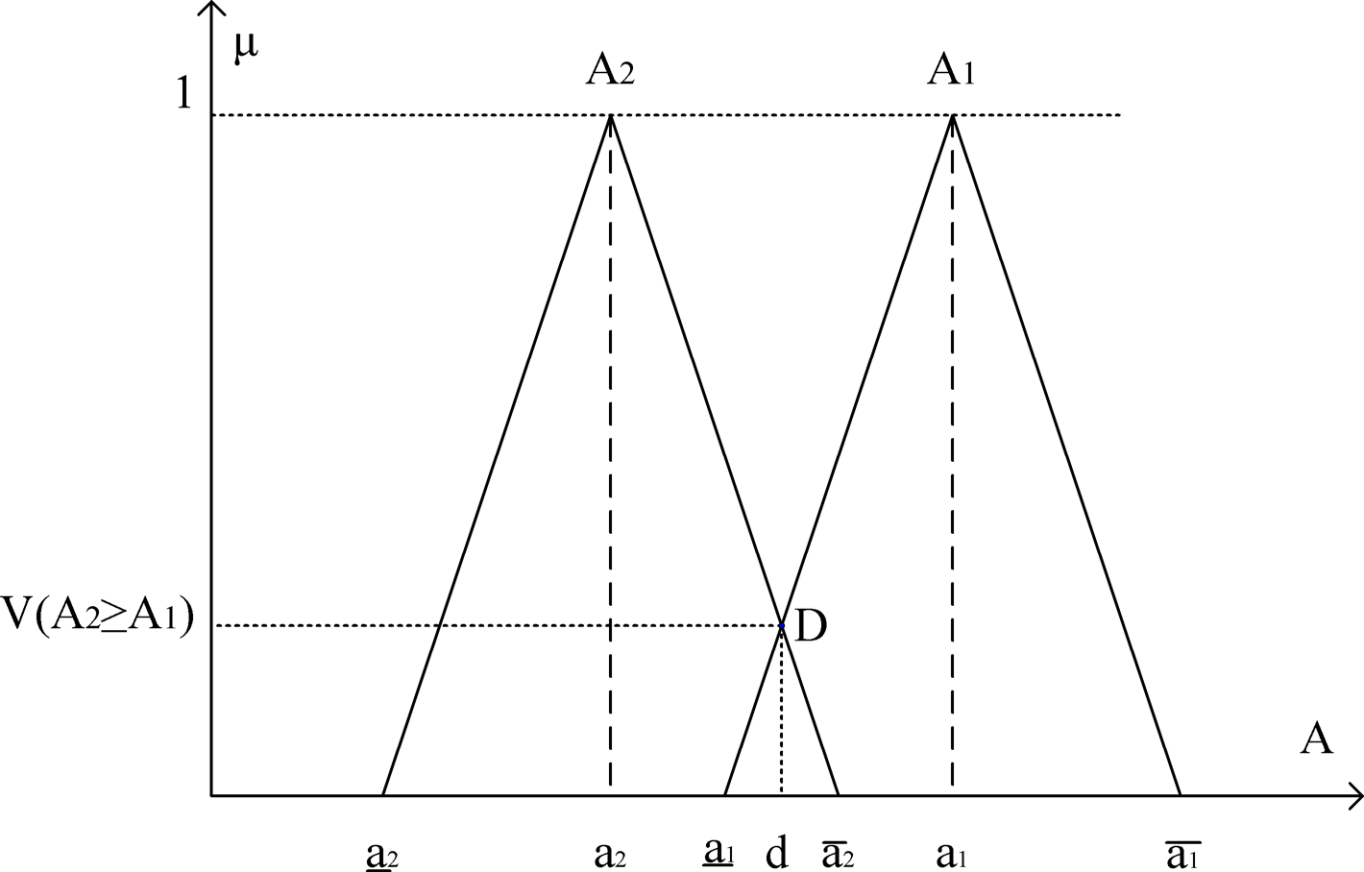
The intersection between TFNs A1 and A2.

D is the highest intersection point between two membership functions $\mu_{A_{1}}$ and $\mu_{A_{2}}$ with ordinate d. The values of *V*(*A*_1_ ≥ *A*_2_) and *V*(*A*_2_ ≥ *A*_1_) are calculated to compare two fuzzy numbers A_1_ and A_2_ (see Fig 3).

**Step 3**: The minimum degree of possibility for a fuzzy number A to be greater than k fuzzy numbers *A*_*i*_(*i* = 1,2,…,*k*) is calculated as follows.

$$V(A\geq A_{1},A_{2},\ldots ,A_{k})=V(A\geq A_{1})\;\text{and}\;(A\geq A_{2})\;\text{and}\ldots \text{and}\;(A\geq A_{k})=\text{min}V(A\geq A_{i}),i=1,2,\ldots ,k$$ (7)

Assume that *d*'(*AT*_*i*_) = min*V*(*A* ≥ *A*_*i*_), for *i* = 1,2,…,*k*. The weighted vector is then

$$W'={\left(d'(AT_{1}),d'(AT_{2}),\ldots ,d'(AT_{n})\right)}^{T},\;\text{where}\;AT_{i}(i=1,2,\ldots ,n)\;\text{comprises n attributes}.$$ (8)

Step 4: The weighted vectors are normalized as follows:

$$W={\left(W_{1},W_{2},\ldots ,W_{n}\right)}^{T},$$ (9)

where W is a crisp or non-fuzzy number.

### Fuzzy ARAS

Fuzzy ARAS is an extension of ARAS that integrates fuzzy logic and was introduced in 2010 by Turskis and Zavadskas [14]. Fuzzy ARAS is a fuzzy discrete MCDM that has proven to be effective in recent years. It has been successfully applied in the fields of economics, transportation, technology, construction and sustainable development. For example, among the significant applications of fuzzy ARAS are the selection of wind farm vendors proposed by Chatterjee and Bose [64], selection of a chief account officer [65, 66], extension of a brand proposed by Zamani *et al*. [67], evaluation of the financial performance of Iranian companies [68, 69], and selection of a deep-water port in the Eastern Baltic Sea [70]. For further understanding, the steps of the fuzzy ARAS procedure can be summarized in Appendix A1.

## Integrated Approach of Fuzzy AHP and Fuzzy ARAS

**Fig 4:**
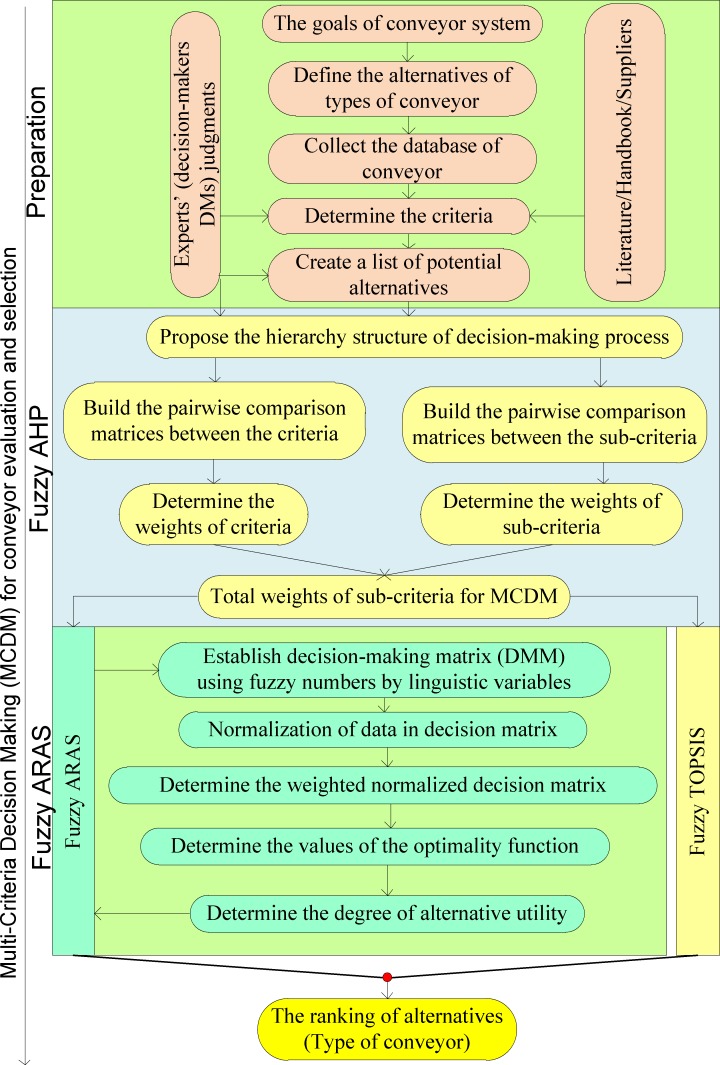
Flowchart of fuzzy AHP and Fuzzy ARAS for conveyor selection.

The flowchart of integration of fuzzy AHP and fuzzy ARAS consists of three phases. First, the database of potential alternatives and criteria are carefully chosen for decision-making based on the literature, handbooks and experience from suppliers and experts. Second, the decision hierarchical structure for conveyor evaluation is established. The pairwise comparison matrices are determined based on the linguistic variables. Then, the weights of criteria and sub-criteria are carried out based on the fuzzy AHP method. Finally, the ranking of conveyor alternatives is determined by the fuzzy ARAS, and its results are validated by fuzzy TOPSIS. The total procedure for evaluation and final selection of conveyor equipment is described in Fig 4.

## Case Study

The proposed framework is implemented in a high-impact research project for conveyor selection in an FMC at one of the university’s manufacturing labs. In this project, the design of the FMC must permit the fabrication of many different part types with various batch sizes in the machining process. The conveyor is used as a material handling system capable of transporting each part type between the CNC machines and other workstations. Moreover, the conveyor assists in the loading and unloading of the different parts at the beginning and end of the machining process. The material handling system in which the conveyor is used is thus critical to the implementation of new FMC systems due to its ability to link together CNC machines, robots, other workstations, and human workers.

Decision making for conveyor selection is complex due to factors such as the facility layout, flexibility, reconfigurability, and the area of the shop floor. Therefore, to choose a suitable alternative, many factors must be considered as qualitative and quantitative criteria in an uncertain manufacturing environment. The relevant criteria for making the right decision quickly are technical, economic, operational, strategic, and ergonomic, as shown in Fig 2. Therefore, careful consideration of the different factors in the MCDM model is essential.

To verify the practical applicability of the proposed model, a case study is performed to evaluate four conveyor alternatives (consisting of rollers and belt conveyors). Four experts in the fields of material handling systems and manufacturing systems are consulted in the multi-criteria evaluation process and assigned as the decision makers (DMs). Information describing the DMs is presented in Table 1. A questionnaire is designed to collect the DMs' judgments (see S1 File). The DMs are then asked to evaluate potential alternatives against the criteria and sub-criteria using linguistic variables, which are presented as triangular fuzzy numbers (Table 2). Linguistic variables are thus used in the questionnaire to convert the measured qualitative factors to fuzzy numbers. The linguistic variables chosen are the commonly used variables just equal (JE), equally important (EI), weakly more important (WMI), strongly more important (SMI), very strongly more important (VSMI), and absolutely more important (AMI). The pairwise comparison matrix between criteria is then formed based on the fuzzy numbers to evaluate the weights using the fuzzy AHP method. Additionally, the decision-making matrix (DMM) is composed using triangular fuzzy numbers, and the weights of the alternatives are determined based on fuzzy ARAS and criteria weights from AHP.

In fuzzy AHP, the comparison matrix is built based on a portion of the questions prepared for the decision makers. For example, a question used in this case study to compare the criteria in Fig 2 is, “The flexibility is how many times as IMPORTANT as the reconfigurability?” The pairwise comparison matrix is formed from the linguistic scale of experts’ importance judgments. Tables 3–8 present the pairwise comparison matrices using the triangular fuzzy numbers from Table 2.

**Table 3:** The pairwise comparison matrix of the criteria with respect to the goals.

	Technical	Cost	Operational	Strategic	Ergonomic
Technical	(1,1,1)	(1/2,2/3,1)	(2/5,1/2,2/3)	(2,5/2,3)	(3/2,2,5/2)
Cost	(1,3/2,2)	(1,1,1)	(1,3/2,2)	(2,5/2,3)	(5/2,3,7/2)
Operational	(3/2,2,5/2)	(1/2,2/3,1)	(1,1,1)	(1,3/2,2)	(1/2,1,3/2)
Strategic	(1/3,2/5,1/2)	(1/3,2/5,1/2)	(1/2,2/3,1)	(1,1,1)	(1,3/2,2)
Ergonomic	(2/5,1/2,2/3)	(2/7,1/3,2/5)	(2/3,1,2)	(1/2,2/3,1)	(1,1,1)

**Table 4:** The pairwise comparison matrix of sub-criteria with respect to the Technical criterion.

	Convenience	Maintainability	Safety	Risk	Repeatability
Convenience	(1,1,1)	(2/7,1/3,2/5)	(2/5,1/2,2/3)	(2/3,1,2)	(2/5,1/2,2/3)
Maintainability	(5/2,3,7/2)	(1,1,1)	(1/2,1,3/2)	(1,3/2,2)	(2/5,1/2,2/3)
Safety	(3/2,2,5/2)	(2/3,1,2)	(1,1,1)	(5/2,3,7/2)	(1,3/2,2)
Risk	(1/2,1,3/2)	(1/2,2/3,1)	(2/7,1/3,2/5)	(1,1,1)	(2/3,1,2)
Repeatability	(3/2,2,5/2)	(3/2,2,5/2)	(1/2,2/3,1)	(1/2,1,3/2)	(1,1,1)

**Table 5:** The pairwise comparison matrix of sub-criteria with respect to the Cost criterion.

	Purchasing cost	Spare parts cost	Set-up and operational cost	Maintenance cost
Purchasing cost	(1,1,1)	(2,5/2,3)	(3/2,2,5/2)	(2,5/2,3)
Spare parts cost	(1/3,2/5,1/2)	(1,1,1)	(3/2,2,5/2)	(1,3/2,2)
Set-up …cost	(2/5,1/2,2/3)	(2/5,1/2,2/3)	(1,1,1)	(1,3/2,2)
Maintenance cost	(1/3,2/5,1/2)	(1/2,2/3,1)	(1/2,2/3,1)	(1,1,1)

**Table 6:** The pairwise comparison matrix of sub-criteria with respect to the Operational criterion.

	Speed	Capacity	Accuracy	Item weight	Item width
Speed	(1,1,1)	(1,3/2,2)	(1,3/2,2)	(2,5/2,3)	(2,5/2,3)
Capacity	(1/2,2/3,1)	(1,1,1)	(2/5,1/2,2/3)	(2,5/2,3)	(2,5/2,3)
Accuracy	(1/2,2/3,1)	(3/2,2,5/2)	(1,1,1)	(5/2,3,7/2)	(5/2,3,7/2)
Item weight	(1/3,2/5,1/2)	(1/3,2/5,1/2)	(2/7,1/3,2/5)	(1,1,1)	(1/2,1,3/2)
Item width	(1/3,2/5,1/2)	(1/3,2/5,1/2)	(2/7,1/3,2/5)	(3/2,1,2)	(1,1,1)

**Table 7:** The pairwise comparison matrix of sub-criteria with respect to the Strategic criterion.

	Flexibility	Guarantee of service	Reconfigurability	Training service
Flexibility	(1,1,1)	(5/2,3,7/2)	(1/2,1,3/2)	(3/2,2,5/2)
Guarantee of service	(2/7,1/3,2/5)	(1,1,1)	(1/3,2/5,1/2)	(1/2,1,3/2)
Reconfigurability	(2/3,1,2)	(2,5/2,3)	(1,1,1)	(3/2,2,5/2)
Training service	(2/5,1/2,2/3)	(2/3,1,2)	(2/5,1/2,2/3)	(1,1,1)

**Table 8:** The pairwise comparison matrix of sub-criteria with respect to the Ergonomic criterion.

	Vibration	Noise	Space for worker	Easy & comfortable to use
Vibration	(1,1,1)	(3/2,2,5/2)	(1/3,2/5,1/2)	(2/5,1/2,2/3)
Noise	(2/5,1/2,2/3)	(1,1,1)	(1/2,2/3,1)	(1/2,2/3,1)
Space for worker	(2,5/2,3)	(1,3/2,2)	(1,1,1)	(1/2,1,3/2)
Easy…to use	(3/2,2,5/2)	(1,3/2,2)	(2/3,1,2)	(1,1,1)

**Fig 5:**
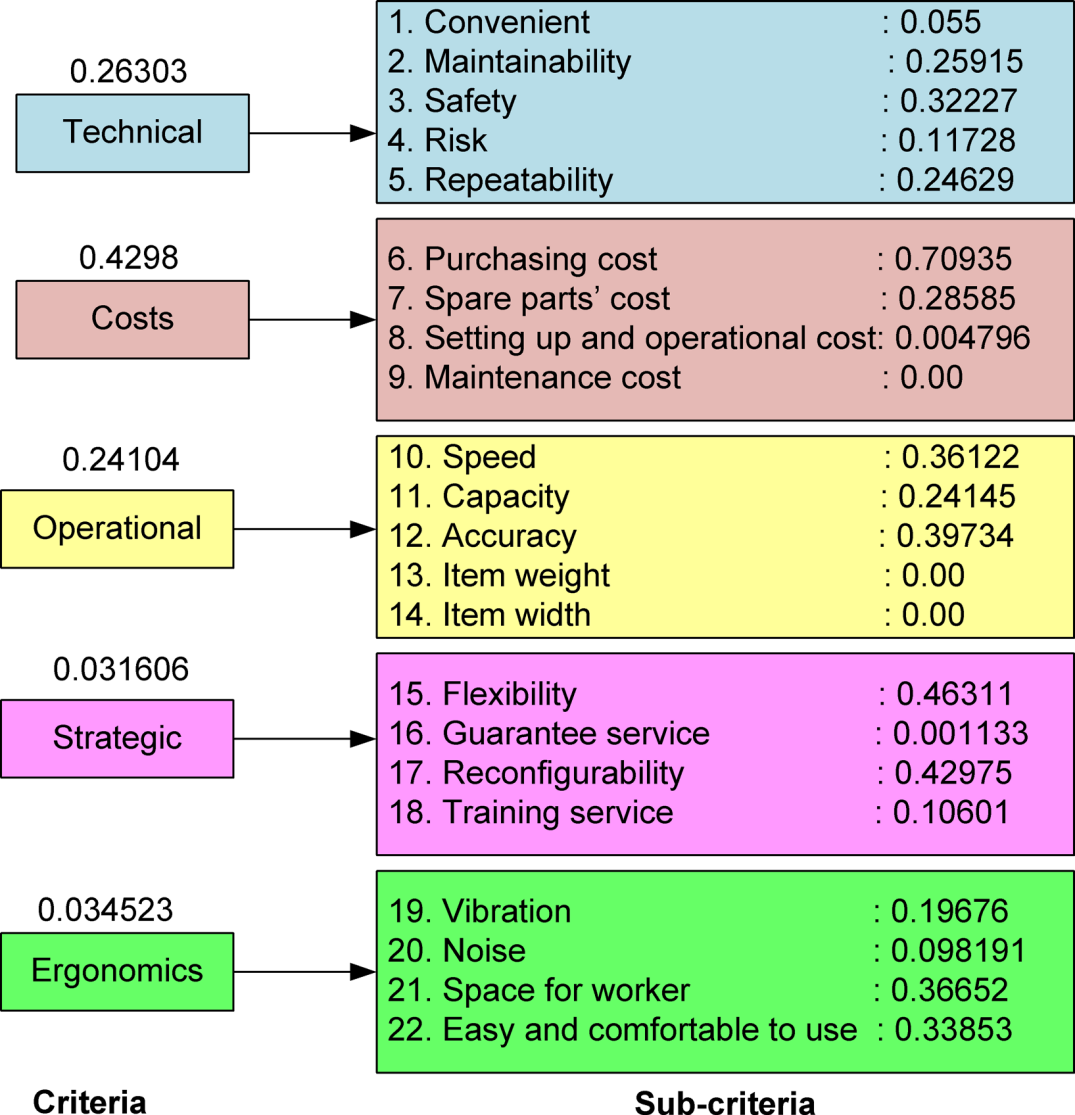
The weights of the criteria and sub-criteria determined based on the fuzzy AHP method.

The weights of the criteria and sub-criteria are calculated based on Eq 1 to Eq 9 of the fuzzy AHP method. A computational program is developed using MATLAB 2013a mathematical software (Mathworks product) on Intel® core™ i5-2410M 2.3GHz, 4GB DDR3 memory with Window 7. The inputs used to determine the weights are the pairwise comparison matrices collected from the survey. The results for the weights of the criteria and sub-criteria are shown in Fig 5.

**Fig 6:**
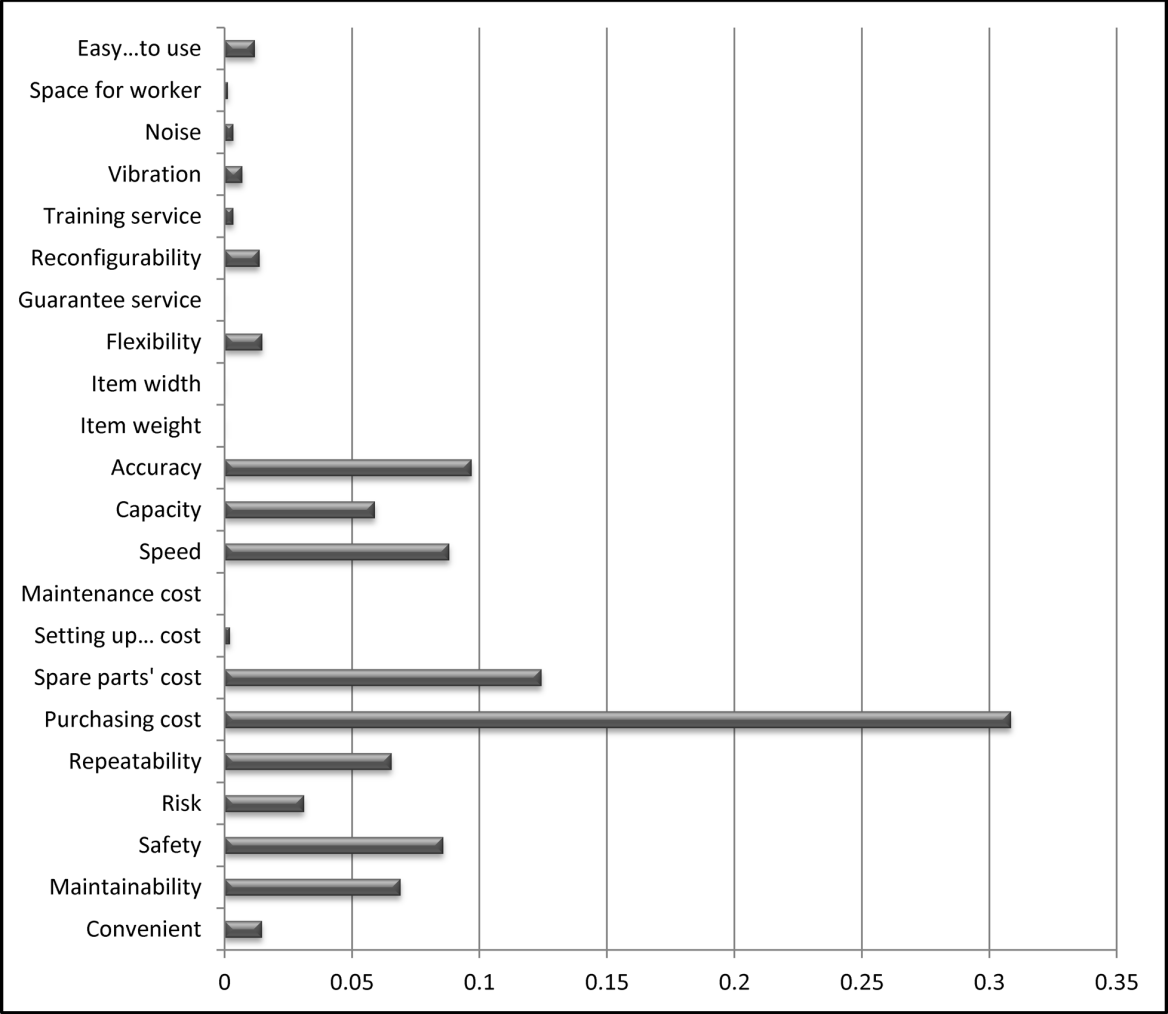
The total weights of the sub-criteria.

The total weights of the sub-criteria for each alternative are determined and normalized in the interval [0,1]. The sub-criteria results are shown in Table 9 and depicted in Fig 6.

**Table 9:** The total weights of the sub-criteria.

Sub-criteria	Weight	Sub-criteria	Weight
Convenience	0.014633333	Accuracy	0.09687834
Maintainability	0.068949604	Item weight	0
Safety	0.085743349	Item width	0
Risk	0.031203587	Flexibility	0.014805701
Repeatability	0.065528065	Guarantee of service	0.00003622
Purchasing cost	0.308391404	Reconfigurability	0.013739176
Spare parts cost	0.124273889	Training service	0.003389157
Set-up… cost	0.002085071	Vibration	0.00687122
Maintenance cost	0	Noise	0.003428905
Speed	0.088071661	Space for worker	0.001279916
Capacity	0.058869671	Easy…to use	0.011821728

The experts use the linguistic terms in Table 10 to evaluate the alternatives with respect to each sub-criterion. The decision matrix is formed as shown in Table 11.

**Table 10:** The linguistic terms used to evaluate the alternatives.

Symbol	Linguistic terms	Triangular fuzzy number
VG	Very Good	(0.9, 1.0, 1.0)
G	Good	(0.7, 0.9, 1.0)
MG	Medium Good	(0.5, 0.7, 0.9)
M	Medium	(0.3, 0.5, 0.7)
MP	Medium Poor	(0.1, 0.3, 0.5)
P	Poor	(0.0, 0.1, 0.3)
VP	Very Poor	(0.0, 0.0, 0.1)

**Table 11:** The decision-making matrix of alternatives with respect to each sub-criterion.

	AT1	AT2	AT3	AT4	Weights
Convenience	G	VG	MG	G	0.014633333
Maintainability	M	VG	MP	G	0.068949604
Safety	VG	G	MG	M	0.085743349
Risk	G	MG	G	MG	0.031203587
Repeatability	VG	VG	VG	VG	0.065528065
Purchasing cost	M	G	MP	MG	0.308391404
Spare parts cost	MP	M	MP	M	0.124273889
Set-up… cost	M	MG	MP	MG	0.002085071
Speed	VG	VG	VG	VG	0.088071661
Capacity	G	G	G	G	0.058869671
Accuracy	MG	G	VG	G	0.09687834
Flexibility	VG	MG	VG	MG	0.014805701
Guarantee of service	VP	M	VP	M	0.00003622
Reconfigurability	VG	G	M	G	0.013739176
Training service	M	G	M	G	0.003389157
Vibration	VG	MG	MG	G	0.00687122
Noise	VG	M	MG	M	0.003428905
Space for worker	G	G	M	G	0.001279916
Easy…to use	MG	MG	M	MG	0.011821728

In Table 12, several sub-criteria (risk, cost, vibration and noise) are desired to be minimal. Therefore, the decision matrix must be altered to implement the data normalization process. Table 13 presents the altered data in the decision matrix based on Eq 14, and Table 14 shows the normalized decision matrix based on Eq 13 and Eq 14.

**Table 12:** The decision-making matrix with triangular fuzzy numbers.

Criteria/Alternatives	AT1	AT2	AT3	AT4	Weights
Convenience	(0.7, 0.9, 1.0)	(0.9, 1.0, 1.0)	(0.5, 0.7, 0.9)	(0.7, 0.9, 1.0)	0.014633333
Maintainability	(0.3, 0.5, 0.7)	(0.9, 1.0, 1.0)	(0.1, 0.3, 0.5)	(0.7, 0.9, 1.0)	0.068949604
Safety	(0.9, 1.0, 1.0)	(0.7, 0.9, 1.0)	(0.5, 0.7, 0.9)	(0.3, 0.5, 0.7)	0.085743349
Risk	(0.7, 0.9, 1.0)	(0.5, 0.7, 0.9)	(0.7, 0.9, 1.0)	(0.7, 0.9, 1.0)	0.031203587
Repeatability	(0.9, 1.0, 1.0)	(0.9, 1.0, 1.0)	(0.9, 1.0, 1.0)	(0.9, 1.0, 1.0)	0.065528065
Purchasing cost	(0.3, 0.5, 0.7)	(0.7, 0.9, 1.0)	(0.1, 0.3, 0.5)	(0.5, 0.7, 0.9)	0.308391404
Spare parts cost	(0.1, 0.3, 0.5)	(0.3, 0.5, 0.7)	(0.1, 0.3, 0.5)	(0.3, 0.5, 0.7)	0.124273889
Set-up… cost	(0.3, 0.5, 0.7)	(0.5, 0.7, 0.9)	(0.1, 0.3, 0.5)	(0.5, 0.7, 0.9)	0.002085071
Speed	(0.9, 1.0, 1.0)	(0.9, 1.0, 1.0)	(0.9, 1.0, 1.0)	(0.9, 1.0, 1.0)	0.088071661
Capacity	(0.7, 0.9, 1.0)	(0.7, 0.9, 1.0)	(0.7, 0.9, 1.0)	(0.7, 0.9, 1.0)	0.058869671
Accuracy	(0.5, 0.7, 0.9)	(0.7, 0.9, 1.0)	(0.9, 1.0, 1.0)	(0.7, 0.9, 1.0)	0.09687834
Flexibility	(0.9, 1.0, 1.0)	(0.5, 0.7, 0.9)	(0.9, 1.0, 1.0)	(0.5, 0.7, 0.9)	0.014805701
Guarantee of service	(0.0, 0.0, 0.1)	(0.3, 0.5, 0.7)	(0.0, 0.0, 0.1)	(0.3, 0.5, 0.7)	0.00003622
Reconfigurability	(0.9, 1.0, 1.0)	(0.7, 0.9, 1.0)	(0.3, 0.5, 0.7)	(0.7, 0.9, 1.0)	0.013739176
Training service	(0.3, 0.5, 0.7)	(0.7, 0.9, 1.0)	(0.3, 0.5, 0.7)	(0.7, 0.9, 1.0)	0.003389157
Vibration	(0.9, 1.0, 1.0)	(0.5, 0.7, 0.9)	(0.5, 0.7, 0.9)	(0.7, 0.9, 1.0)	0.00687122
Noise	(0.9, 1.0, 1.0)	(0.3, 0.5, 0.7)	(0.5, 0.7, 0.9)	(0.3, 0.5, 0.7)	0.003428905
Space for worker	(0.7, 0.9, 1.0)	(0.7, 0.9, 1.0)	(0.3, 0.5, 0.7)	(0.7, 0.9, 1.0)	0.001279916
Easy…to use	(0.5, 0.7, 0.9)	(0.5, 0.7, 0.9)	(0.3, 0.5, 0.7)	(0.5, 0.7, 0.9)	0.011821728

**Table 13:** The changed decision-making matrix.

	AT0	AT1	AT2	AT3	AT4	Total
Convenience	1	(0.7, 0.9, 1.0)	(0.9, 1.0, 1.0)	(0.5, 0.7, 0.9)	(0.7, 0.9, 1.0)	(2.8,3.5,3.9)
Maintainability	1	(0.3, 0.5, 0.7)	(0.9, 1.0, 1.0)	(0.1, 0.3, 0.5)	(0.7, 0.9, 1.0)	(2,2.7,3.2)
Safety	1	(0.9, 1.0, 1.0)	(0.7, 0.9, 1.0)	(0.5, 0.7, 0.9)	(0.3, 0.5, 0.7)	(2.4,3.1,3.6)
Risk*	2	(1.43, 1.11, 1.0)	(2, 1.43, 1.11)	(1.43,1.11, 1.0)	(1.43, 1.11, 1.0)	(6.29,4.76,4.11)
Repeatability	1	(0.9, 1.0, 1.0)	(0.9, 1.0, 1.0)	(0.9, 1.0, 1.0)	(0.9, 1.0, 1.0)	(3.6,4,4)
Purchasing cost*	10	(3.33, 2, 1.43)	(1.43, 1.11, 1.0)	(10, 3.33, 2)	(2,1.43,1.11)	(16.76,7.87,5.54)
Spare parts cost*	10	(10, 3.33,2)	(3.33, 2, 1.43)	(10, 3.33,2)	(3.33,2,1.43)	(26.66,10.66,6.86)
Set-up… cost*	10	(3.33, 2, 1.43)	(2,1.43,1.11)	(10,3.33,2)	(2, 1.43, 1.11)	(17.33,8.19,5.65)
Speed	1	(0.9, 1.0, 1.0)	(0.9, 1.0, 1.0)	(0.9, 1.0, 1.0)	(0.9, 1.0, 1.0)	(3.6,4,4)
Capacity	1	(0.7, 0.9, 1.0)	(0.7, 0.9, 1.0)	(0.7, 0.9, 1.0)	(0.7, 0.9, 1.0)	(2.8,3.6,4)
Accuracy	1	(0.5, 0.7, 0.9)	(0.7, 0.9, 1.0)	(0.9, 1.0, 1.0)	(0.7, 0.9, 1.0)	(2.8,3.5,3.9)
Flexibility	1	(0.9, 1.0, 1.0)	(0.5, 0.7, 0.9)	(0.9, 1.0, 1.0)	(0.5, 0.7, 0.9)	(2.8,3.4,3.8)
Guarantee of service	0.7	(0.0, 0.0, 0.1)	(0.3, 0.5, 0.7)	(0.0, 0.0, 0.1)	(0.3, 0.5, 0.7)	(0.6,1,1.6)
Reconfigurability	1	(0.9, 1.0, 1.0)	(0.7, 0.9, 1.0)	(0.3, 0.5, 0.7)	(0.7, 0.9, 1.0)	(2.6,3.3,3.7)
Training service	1	(0.3, 0.5, 0.7)	(0.7, 0.9, 1.0)	(0.3, 0.5, 0.7)	(0.7, 0.9, 1.0)	(2,2.8,3.4)
Vibration*	2	(1.11, 1.0, 1.0)	(2, 1.43, 1.11)	(2, 1.43, 1.11)	(1.43, 1.11, 1.0)	(6.54,4.97,4.22)
Noise*	3.33	(1.11, 1.0, 1.0)	(3.33,2,1.43)	(2, 1.43, 1.11)	(3.33,2,1.43)	(9.77,6.43,4.97)
Space for worker	1	(0.7, 0.9, 1.0)	(0.7, 0.9, 1.0)	(0.3, 0.5, 0.7)	(0.7, 0.9, 1.0)	(2.4,3.2,3.7)
Easy…to use	0.9	(0.5, 0.7, 0.9)	(0.5, 0.7, 0.9)	(0.3, 0.5, 0.7)	(0.5, 0.7, 0.9)	(1.8,2.6,3.4)

* For the criteria with preferred minimum values

**Table 14:** The normalized decision-making matrix.

	AT0	AT1	AT2	AT3	AT4	Total
Convenience	(0.256, 0.286, 0.357)	(0.179, 0.257, 0.357)	(0.231, 0.286, 0.357)	(0.128, 0.2, 0.321)	(0.179, 0.257, 0.357)	(2.8,3.5,3.9)
Maintainability	(0.313,0.37,0.5)	(0.094, 0.185, 0.35)	(0.281, 0.37, 0.5)	(0.031, 0.111, 0.25)	(0.219, 0.333, 0.5)	(2,2.7,3.2)
Safety	(0.278,0.323,0.417)	(0.25, 0.323, 0.417)	(0.194, 0.29, 0.417)	(0.139, 0.226, 0.375)	(0.083, 0.161, 0.292)	(2.4,3.1,3.6)
Risk*	(0.487,0.42,0.318)	(0.348, 0.233, 0.159)	(0.487, 0.3, 0.176)	(0.348,0.2330.159)	(0.348, 0.233, 0.159)	(6.29,4.76,4.11)
Repeatability	(0.25,0.25,0.278)	(0.225, 0.25, 0.278)	(0.225, 0.25, 0.278)	(0.225, 0.25, 0.278)	(0.225, 0.25, 0.278)	(3.6,4,4)
Purchasing cost*	(1.8,1.27,0.597)	(0.601,0.254,0.085)	(0.258, 0.141,0.06)	(1.8,0.423,0.119)	(0.361,0.182,0.066)	(16.76,7.87,5.54)
Spare parts cost*	(1.46,0.938,0.075)	(1.46,0.312,0.075)	(0.485, 0.188, 0.054)	(1.46, 0.312,0.075)	(0.485,0.188,0.054)	(26.66,10.66,6.86)
Set-up… cost*	(1.77,1.22,0.577)	(0.589,0.244,0.083)	(0.354,0.175,0.064)	(1.77,0.41,0.115)	(0.354,0.175,0.064)	(17.33,8.19,5.65)
Speed	(0.25,0.25,0.278)	(0.225, 0.25, 0.278)	(0.225, 0.25, 0.278)	(0.225, 0.25, 0.278)	(0.225, 0.25, 0.278)	(3.6,4,4)
Capacity	(0.25,0.278,0.357)	(0.175, 0.25,0.357)	(0.175, 0.25,0.357)	(0.175, 0.25,0.357)	(0.175, 0.25,0.357)	(2.8,3.6,4)
Accuracy	(0.256,0.286,0.357)	(0.128, 0.2, 0.321)	(0.179, 0.257,0.357)	(0.23,0.286,0.357)	(0.179, 0.257,0.357)	(2.8,3.5,3.9)
Flexibility	(0.263,0.294,0.357)	(0.237,0.294,0.357)	(0.132,0.206,0.321)	(0.237,0.294,0.357)	(0.132,0.206,0.321)	(2.8,3.4,3.8)
Guarantee of service	(0.438,0.7,1.167)	(0.0, 0.0, 0.167)	(0.188, 0.5, 0.167)	(0.0, 0.0, 0.167)	(0.188, 0.5, 0.167)	(0.6,1,1.6)
Reconfigurability	(0.27,0.303,0.384)	(0.243,0.303,0.384)	(0.189, 0.273, 0.384)	(0.081,0.152,0.269)	(0.189, 0.273, 0.384)	(2.6,3.3,3.7)
Training service	(0.294,0.357,0.5)	(0.088, 0.179, 0.35)	(0.206,0.321,0.5)	(0.088, 0.179, 0.35)	(0.206,0.321,0.5)	(2,2.8,3.4)
Vibration*	(0.474,0.403,0.306)	(0.263,0.201,0.153)	(0.474,0.288,0.17)	(0.474,0.288,0.17)	(0.34,0.223,0.153)	(6.54,4.97,4.22)
Noise*	(0.67,0.518,0.341)	(0.223,0.156,0.102)	(0.67,0.311,0.146)	(0.402,0.223,0.114)	(0.67,0.311,0.146)	(9.77,6.43,4.97)
Space for worker	(0.27,0.313,0.417)	(0.189, 0.281,0.417)	(0.189, 0.281,0.417)	(0.081,0.156,0.292)	(0.189, 0.281,0.417)	(2.4,3.2,3.7)
Easy…to use	(0.265,0.346,0.5)	(0.147,0.269,0.5)	(0.147,0.269,0.5)	(0.088,0.192,0.389)	(0.147,0.269,0.5)	(1.8,2.6,3.4)

* For the criteria with preferred minimum values

Using Eq 15 and Eq 16, the weighted normalized decision-making matrix is calculated, and the values of the optimality function are determined based on Eq 17. The defuzzification process uses Eq 18, and the degree of alternative utility is determined from Eq 19. The results are presented in Table 15.

**Table 15:** The weighted normalized decision-making matrix.

	AT0	AT1	AT2	AT3	AT4
Convenience	(0.003746, 0.0042,0.00522)	(0.00262, 0.00376,0.00522)	(0.0034, 0.0042, 0.0052)	(0.0019,0.0029,0.0047)	(0.0026,0.0038,0.0052)
Maintainability	(0.0216,0.0255,0.0345)	(0.0065, 0.0128, 0.0241)	(0.0194, 0.0255, 0.0345)	(0.0021, 0.0077, 0.0172)	(0.0151, 0.023, 0.0345)
Safety	(0.0238,0.0277,0.0358)	(0.0214, 0.0277, 0.0358)	(0.0166, 0.0249, 0.0358)	(0.0119, 0.0194, 0.0322)	(0.0071, 0.0138, 0.025)
Risk*	(0.0152,0.0131,0.0099)	(0.0109, 0.0073, 0.005)	(0.0152, 0.0094, 0.0055)	(0.0109,0.0073,0.005)	(0.0152, 0.0073, 0.005)
Repeatability	(0.0164,0.0164,0.0182)	(0.0147, 0.0164, 0.0182)	(0.0147, 0.0164, 0.0182)	(0.0147, 0.0164, 0.0182)	(0.0147, 0.0164, 0.0182)
Purchasing cost*	(0.555,0.392,0.184)	(0.185,0.0783,0.0262)	(0.0796, 0.0435,0.0185)	(0.555,0.13,0.0367)	(0.111,0.056,0.0204)
Spare parts cost*	(0.181,0.1166,0.0093)	(0.181,0.0388,0.0093)	(0.0603, 0.0234, 0.0067)	(0.181,0.0388,0.0093)	(0.0603, 0.0234, 0.0067)
Set-up… cost*	(0.0037,0.0025, 0.0012)	(0.0012,0.0005,0.0002)	(0.0007,0.0004,0.0001)	(0.0037,0.0009,0.0002)	(0.0007,0.0004,0.0001)
Speed	(0.022,0.022,0.0245)	(0.0198, 0.022, 0.0245)	(0.0198, 0.022, 0.0245)	(0.0198, 0.022, 0.0245)	(0.0198, 0.022, 0.0245)
Capacity	(0.0147,0.0164,0.021)	(0.0103, 0.0147,0.021)	(0.0103, 0.0147,0.021)	(0.0103, 0.0147,0.021)	(0.0103, 0.0147,0.021)
Accuracy	(0.0248,0.0277,0.0346)	(0.0124, 0.0194, 0.0311)	(0.0173, 0.0249,0.0346)	(0.0223,0.0277,0.0346)	(0.0173, 0.0249,0.0346)
Flexibility	(0.0039,0.0044,0.0053)	(0.0035,0.0044,0.0053)	(0.002,0.0031,0.0048)	(0.0035,0.0044,0.0053)	(0.002,0.0031,0.0048)
Guarantee of service	(0.000016,0.000025,0.000042)	(0.0, 0.0, 0.00001)	(0.00001, 0.00002, 0.00001)	(0.0, 0.0, 0.00001)	(0.00001, 0.00002, 0.00001)
Reconfigurability	(0.0037,0.0042,0.0053)	(0.0033,0.0042,0.0053)	(0.0026, 0.0038, 0.0053)	(0.0011,0.0021,0.0037)	(0.0026, 0.0038, 0.0053)
Training service	(0.001,0.0012,0.0017)	(0.0003, 0.0006, 0.0012)	(0.0007,0.0011,0.0017)	(0.0003, 0.0006, 0.0012)	(0.0007,0.0011,0.0017)
Vibration*	(0.0033,0.0028,0.0021)	(0.0018,0.0014,0.0011)	(0.0033,0.002,0.0012)	(0.0033,0.002,0.0012)	(0.0023,0.0015,0.0011)
Noise*	(0.0023,0.0018,0.0012)	(0.0008,0.0005,0.0004)	(0.0023,0.0011,0.0005)	(0.0014,0.0008,0.0004)	(0.0023,0.0011,0.0005)
Space for worker	(0.0003,0.0004,0.0005)	(0.0002, 0.0004,0.0005)	(0.0002, 0.0004,0.0005)	(0.0001,0.002,0.0004)	(0.0002, 0.0004,0.0005)
Easy…to use	(0.0031,0.0041,0.0059)	(0.0017,0.0032,0.0059)	(0.0017,0.0032,0.0059)	(0.001,0.0023,0.0046)	(0.0017,0.0032,0.0059)
Si	(0.899562,0.683,0.4)	(0.47742, 0.25636,0.22033)	(0.27011, 0.224,0.22451)	(0.8443,0.302,0.22)	(0.2859,0.21992,0.215)
Si	0.661	0.318	0.2395	0.4554	0.2403
Ki = Si/So	1	0.481	0.362	0.689	0.364

**Fig 7:**
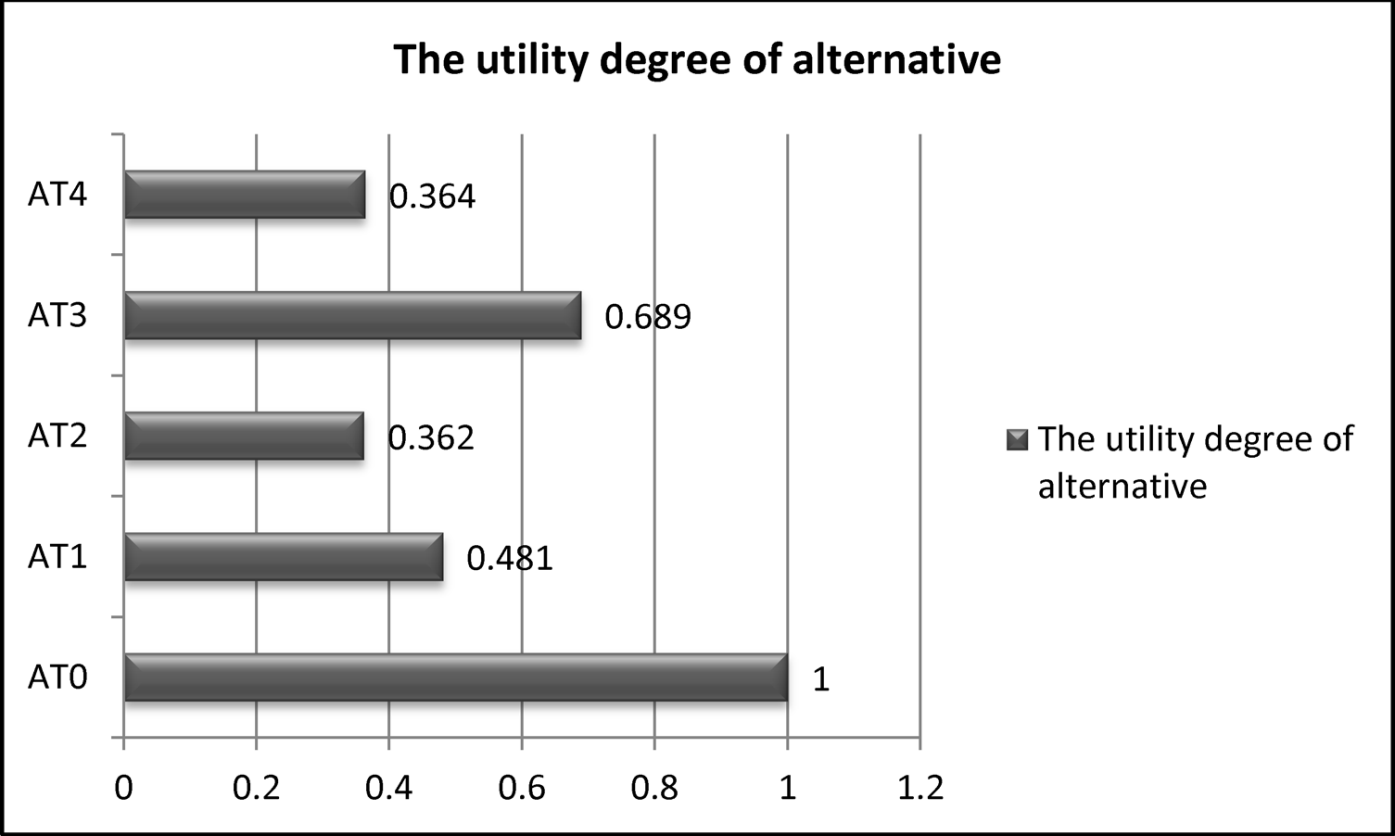
The utility degree of the alternatives.

**Fig 8:**
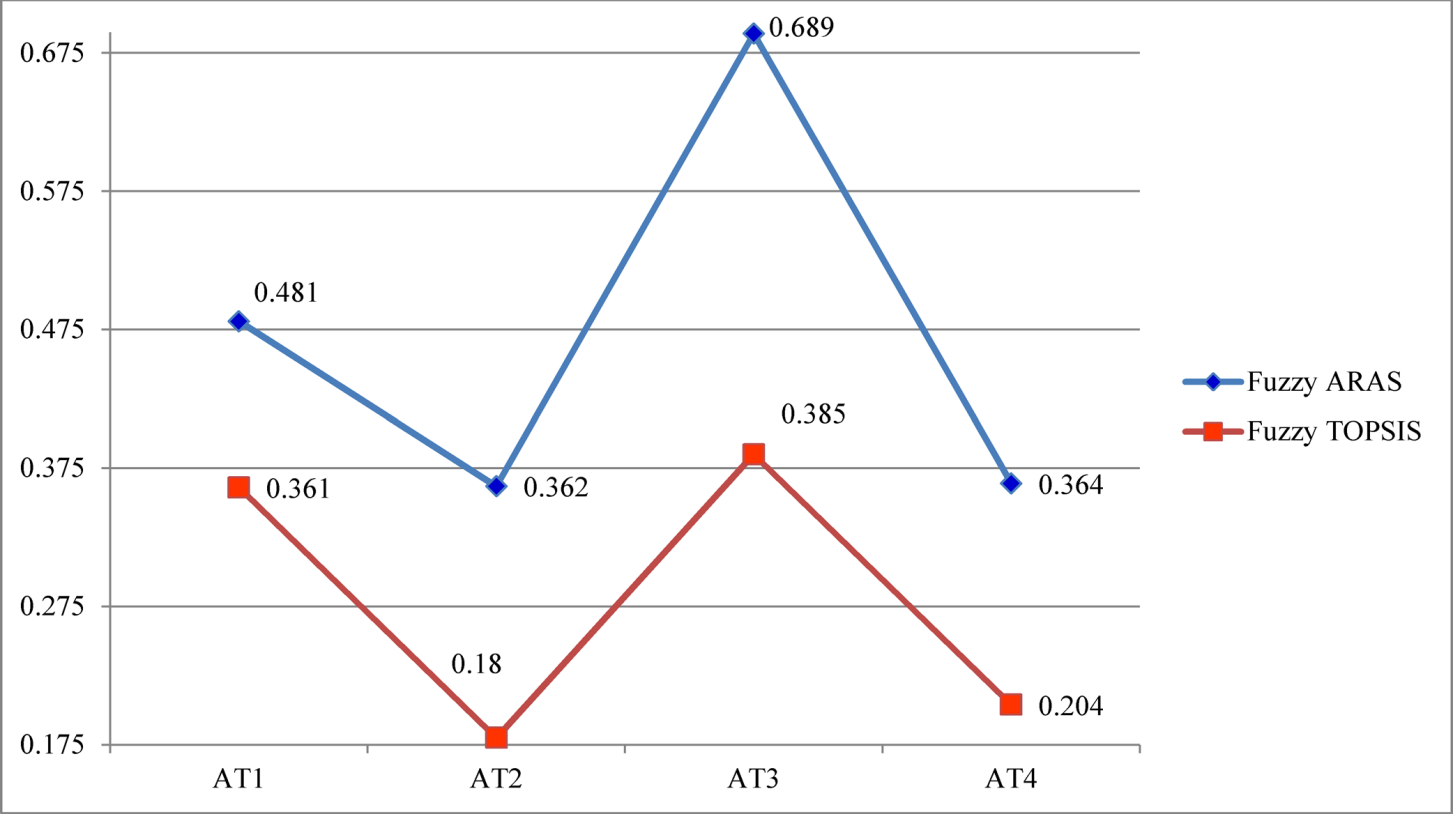
Comparative analysis of ranking between fuzzy ARAS and fuzzy TOPSIS.

As depicted in Fig 7, the ranking sequence is *AT*3 ≻ *AT*1 ≻ *AT*4 ≻ *AT*2. This ranking is similar to the ranking resulting from the TOPSIS approach [71]. Moreover, the results are validated by comparing with the ranking obtained from fuzzy TOPSIS methodology [72, 73, 74]. These results of fuzzy TOPSIS are described by PIS (positive ideal solution), NIS (negative ideal solution) and closeness coefficient (cc) as weights of alternatives (see Table 16, Fig 8). Table 16 shows that the ranking of two methods (fuzzy ARAS and fuzzy TOPSIS) is an evidence for the validation of proposed framework in this paper. Fig 8 shows that AT3 is the best alternative because its degree of alternative utility is the highest.

**Table 16:** Comparison between fuzzy ARAS and fuzzy TOPSIS.

	Weight of Alternatives					
Method	Fuzzy ARAS		Fuzzy TOPSIS			
Alternatives	Weight	Ranking	PIS	NIS	Weight	Ranking
AT1	0.481	2	0.282	0.159	0.361	2
AT2	0.362	4	0.704	0.155	0.18	4
AT3	0.689	1	0.521	0.326	0.385	1
AT4	0.364	3	0.673	0.173	0.204	3

## Conclusion and Future Work

In a practical manufacturing environment, the MCDM for selecting an alternative is a complicated process because it involves many qualitative and quantitative criteria, and the information collected is usually vague and imprecise. In this case study, the conveyor is selected based on a multi-criteria evaluation using experts' judgments. The experts choose the conveyor alternative using the proposed framework that integrated fuzzy set, AHP and fuzzy ARAS. It is the main contribution of this paper. There is no paper solving the problem of selecting the conveyor using this approach with considerations of some new criteria. In particular a fuzzy number is used to convert the qualitative information into crisp data, and the steps of fuzzy AHP are used to determine the weights of the criteria and sub-criteria without considering the data consistency. This approach is helpful to aid the decision-makers because of the potential benefits. Fuzzy AHP consisted of many outstanding advantages in handling the uncertainty and provided an effective way to describe the quantitative and qualitative data. Moreover, this method did not consider the consistency ratio, so it reduced the number of experts' judgment needed to collect in decision-making. Fuzzy ARAS is used to determine the ranking of the alternatives and also to reduce the matrix of pairwise comparisons between alternatives from the fuzzy AHP when the number of alternatives is large. It effectively handled the uncertain information and achieved the final selection of the most appropriate conveyor alternative. Therefore, the combination of the most powerful feature of fuzzy AHP and fuzzy ARAS required less computational power in making decision more quickly and accurate. Especially, this integrated approach is effective in the case of large number of alternative. It should be noted that the new factors considered in this study include ergonomics (i.e., vibration, noise, and space for the worker) and reconfigurability. The weightage of the purchasing cost is the highest. This is entirely consistent with the psychology of customers in decision-making to select the desired product in practice. This shows that the cost of equipment is extremely important in decision making for evaluation and selection. The ergonomics criterion also takes into account the working space for the worker and the working conditions in the manufacturing environment. The weight of ergonomics factor is relative low in evaluating and selecting the conveyor equipment. This is a significant finding because it demonstrated the developing countries were not really interested in proper investment to improve the working environment and health of the workforce. The reconfigurability criterion represents the capability of restructuring the conveyor according to the manufacturing demands of SMEs. Safety, maintainability, accuracy, speed, and capacity are critical criteria used to ensure proper equipment operation for the requirements of SMEs. This highlighted that the manufacturing SMEs have paid the right attention in selection decision.

A limitation of this method is that it depends on the experience of the experts and the quality of the statements from the experts’ review. The hierarchical structure between the criteria and sub-criteria does not yet consider mutual relationships (i.e., interdependence) as in the ANP method. However, the method has the advantage of enabling a quick decision in a competitive manufacturing environment. The numerical example also demonstrates that the proposed model can achieve effective and flexible decisions in choosing a conveyor as well as other equipment. In the future, this integration could be applied to the selection of other devices in engineering, business processes, advanced manufacturing technologies, and decisions at management levels. Besides, the AHP methods can be extended with D numbers [75] and its applications are potential studies in uncertain MCDM environment. Additionally, the expansion of fuzzy AHP with methods such as PROMETHEE, VIKOR, SAW, COPRAS, ELECTRE and TOPSIS is also promising for the analysis of the sensitivity of the model.

## Appendix

### A1. The steps of the fuzzy ARAS procedure:

**Step 1**: Formation of the decision-making matrix (DMM), with m alternatives and n criteria.

$${\tilde{A}}_{m\times n}=\left[\begin{array}{lllll} {\tilde{a}}_{01} & \cdots & {\tilde{a}}_{0j} & \cdots & {\tilde{a}}_{0n}\\ \vdots & \ddots & \vdots & \ddots & \vdots \\ {\tilde{a}}_{i1} & \cdots & {\tilde{a}}_{ij} & \cdots & {\tilde{a}}_{in}\\ \vdots & \ddots & \vdots & \ddots & \vdots \\ {\tilde{a}}_{m1} & \cdots & {\tilde{a}}_{mj} & \cdots & {\tilde{a}}_{mn} \end{array}\right],\;\text{where}\;i=0,1,2,\ldots ,m;j=1,2,\ldots ,n$$ (10)

where ${\tilde{a}}_{ij}$ is a fuzzy number instantiating the performance of alternative *i* to criterion *j* and ${\tilde{a}}_{0j}$ is the optimal value of criterion *j*. The symbol ‘~’ indicates a fuzzy set.

If the optimal value of a criterion is unknown in advance, then we can define it as follows:

$$\begin{array}{l} {\tilde{a}}_{0j}=\overset{m}{\underset{i=1}{\text{max}}}\;a_{ij},\;\text{if}\;\overset{m}{\underset{i=1}{\text{max}}}\;a_{ij}\;\text{is preferable},\;\text{and}\\ {\tilde{a}}_{0j}=\overset{m}{\underset{i=1}{\text{min}}}\;a_{ij},\;\text{if}\;\overset{m}{\underset{i=1}{\text{min}}}\;a_{ij}\;\text{is preferable} \end{array}$$ (11)

**Step 2**: Normalization of the DMM.

$${\tilde{\bar{A}}}_{m\times n}=\left[\begin{array}{lllll} {\tilde{\bar{a}}}_{01} & \cdots & {\tilde{\bar{a}}}_{0j} & \cdots & {\tilde{\bar{a}}}_{0n}\\ \vdots & \ddots & \vdots & \ddots & \vdots \\ {\tilde{\bar{a}}}_{i1} & \cdots & {\tilde{\bar{a}}}_{ij} & \cdots & {\tilde{\bar{a}}}_{in}\\ \vdots & \ddots & \vdots & \ddots & \vdots \\ {\tilde{\bar{a}}}_{m1} & \cdots & {\tilde{\bar{a}}}_{mj} & \cdots & {\tilde{\bar{a}}}_{mn} \end{array}\right],\;\text{where}\;i=0,1,2,\ldots ,m;j=1,2,\ldots ,n$$ (12)

For criteria with preferred maximum values, the normalization is performed as follows:

$${\tilde{\bar{a}}}_{ij}=\frac{{\tilde{a}}_{ij}}{\sum_{i=0}^{m}{\tilde{a}}_{ij}}$$ (13)

For criteria with preferred minimum values, the normalization is determined using a two-stage procedure:

$${\tilde{a}}_{ij}=\frac{1}{{\tilde{a}}_{ij}^{*}};{\tilde{\bar{a}}}_{ij}=\frac{{\tilde{a}}_{ij}}{\sum_{i=0}^{m}{\tilde{a}}_{ij}}$$ (14)

**Step 3**: Determination of the weighted normalized decision-making matrix $\hat{A}$.

$${\tilde{\hat{A}}}_{m\times n}=\left[\begin{array}{lllll} {\tilde{\hat{a}}}_{01} & \cdots & {\tilde{\hat{a}}}_{0j} & \cdots & {\tilde{\hat{a}}}_{0n}\\ \vdots & \ddots & \vdots & \ddots & \vdots \\ {\tilde{\hat{a}}}_{i1} & \cdots & {\tilde{\hat{a}}}_{ij} & \cdots & {\tilde{\hat{a}}}_{in}\\ \vdots & \ddots & \vdots & \ddots & \vdots \\ {\tilde{\hat{a}}}_{m1} & \cdots & {\tilde{\hat{a}}}_{mj} & \cdots & {\tilde{\hat{a}}}_{mn} \end{array}\right],\;\text{where}\;i=0,1,2,\ldots ,m;j=1,2,\ldots ,n$$ (15)

The normalized weighted values of the criteria are determined as follows:

$${\tilde{\hat{a}}}_{ij}={\tilde{\bar{a}}}_{ij}\times w_{j};\;i=0,1,2,\ldots ,m$$ (16)

where $w_{j},{\bar{a}}_{ij}$ are the weights and the normalized value of the criterion j.

**Step 4**: Determination of the values of the optimality function.

$${\tilde{S}}_{i}=\sum_{j=1}^{n}{\tilde{\hat{a}}}_{ij};\;\text{with}\;i=1,2,\ldots ,n$$ (17)

where ${\tilde{S}}_{i}$ is the value of the optimality function for the i^th^ alternative. The largest value represents the best alternative, and the smallest value represents the worst. Therefore, the greater the value of the optimality function ${\tilde{S}}_{i}$, the more effective the alternative. Because the values ${\tilde{S}}_{i}$ used to rank the alternatives are fuzzy numbers, we need to defuzzify ${\tilde{S}}_{i}$ using the center-of-area formula:

$$S_{i}=\frac{1}{3}(S_{i\alpha }+S_{i\beta }+S_{i\gamma })$$ (18)

**Step 5**: Calculation of the degree of alternative utility by comparing each variant S_i_ with the ideal, S_0_.

$$K_{i}=\frac{S_{i}}{S_{0}};\;{\text{where S}}_{i},S_{0}\;\text{are the optimal values of criteria}$$ (19)

Each *K*_*i*_ is the utility of alternative *i*, with a value in the interval [0, 1]. The *K*_*i*_ can be arranged in an increasing sequence, i.e., in the desired order of precedence. The complex relative efficiency of each reasonable alternative can be determined according to the utility function values.

## Supporting Information

S1 File Questionnaire design for multi-criteria decision making in conveyor evaluation and selection (PDF)
