# Supplementary material for: An Integrated MCDM Model for Conveyor Equipment Evaluation and Selection in an FMC Based on a Fuzzy AHP and Fuzzy ARAS in the Presence of Vagueness
Source: PLoS One. 2016 Apr 12;11(4):e0153222. doi: 10.1371/journal.pone.0153222 (PMC4829176; doi:10.1371/journal.pone.0153222)
Supplement: S1 File — (PDF) [file pone.0153222.s001.pdf]

S1 File. Questionnaire design for multi-criteria decision-making in conveyor evaluation and selection

The purpose of the questionnaire design is to determine the weights/priorities of the selected criteria for multi-criteria decision-making (MCDM) process in evaluating and selecting the conveyor system of a Flexible Manufacturing Cell (FMC), satisfying the desired requirements of manufacturing from Small and Medium Enterprise's (SME). The result is used for the academic and reference in Malaysia. The questionnaire should be completed by the experts or operators understanding the materials handling equipment (MHE). The following questions refer a questionnaire hierarchical structure to determine the importance of the criteria and the weights/priorities of alternatives by putting check marks on the pair-wise comparison matrices. An example is shown as follows.

No.	Linguistic scale for importance	Triangular fuzzy scale
1	Just equal (JE)	(1,1,1)
2	Equally importance (EI)	(1/2,1,3/2)
3	Weakly more important (WMI)	(1,3/2,2)
4	Strongly more important (SMI)	(3/2,2,5/2)
5	Very strongly more important (VSMI)	(2,5/2,3)
6	Absolutely more important (AMI)	(5/2,3,7/2)

Question: How important is Criterion 1 when it is compared with Criterion 2 for the conveyor selection? If Criterion 1 is more important than Criterion 2, please mark the scale number on the left. If Criterion 2 is more important than Criterion 1, please mark on the right. Please choose 1, 2, 3, 4, 5, and 6 for important level.

Criterion 1	Scale number for pair-wise comparisons											Criterion 2
Technical	6	5	4	3	2	**1**	2	3	4	5	6	Costs
Technical	6	5	4	3	2	**1**	2	3	4	5	6	Operational
Technical	6	5	4	3	2	**1**	2	3	4	5	6	Strategic
Technical	6	5	4	3	2	**1**	2	3	4	5	6	Ergonomics
Costs	6	5	4	3	2	**1**	2	3	4	5	6	Operational
Costs	6	5	4	3	2	**1**	2	3	4	5	6	Strategic
Costs	6	5	4	3	2	**1**	2	3	4	5	6	Ergonomics
Operational	6	5	4	3	2	**1**	2	3	4	5	6	Strategic
Operational	6	5	4	3	2	**1**	2	3	4	5	6	Ergonomics
Strategic	6	5	4	3	2	**1**	2	3	4	5	6	Ergonomics

Section 2: Questionnaire form used to compare the sub-criteria in each criteria group. For instance, the questionnaire for the target < **Technical** >

Question: For the target "**Technical**", how important? What is the influence level of the sub-criteria 1 with respect to the satisfaction of the target when it is compared with the sub-criteria 2? If sub-criterion 1 is more important than sub-criterion 2, please mark the scale number (1, 2, 3, 4, 5, and 6) on the left. If sub-criterion 2 is more important than sub-criterion 1, please mark on the right.

Sub-criterion 1	Scale number for pair-wise comparisons											Sub-criterion 2
Convenient	6	5	4	3	2	**1**	2	3	4	5	6	Maintainability
Convenient	6	5	4	3	2	**1**	2	3	4	5	6	Safety
Convenient	6	5	4	3	2	**1**	2	3	4	5	6	Risk
Convenient	6	5	4	3	2	**1**	2	3	4	5	6	Repeatability
Maintainability	6	5	4	3	2	**1**	2	3	4	5	6	Safety
Maintainability	6	5	4	3	2	**1**	2	3	4	5	6	Risk
Maintainability	6	5	4	3	2	**1**	2	3	4	5	6	Repeatability
Safety	6	5	4	3	2	**1**	2	3	4	5	6	Risk
Safety	6	5	4	3	2	**1**	2	3	4	5	6	Repeatability
Risk	6	5	4	3	2	**1**	2	3	4	5	6	Repeatability

………

Section 3: Evaluating the conveyor equipment based on the experts' judgments using the linguistic terms.

**Symbol**	**Linguistic terms**	**Triangular Fuzzy number**
VG	Very Good	(0.9, 1.0, 1.0)
G	Good	(0.7, 0.9, 1.0)
MG	Medium Good	(0.5, 0.7, 0.9)
M	Medium	(0.3, 0.5, 0.7)
MP	Medium Poor	(0.1, 0.3, 0.5)
P	Poor	(0.0, 0.1, 0.3)
VP	Very Poor	(0.0, 0.0, 0.1)

Questions: How to evaluate the sub-criteria for each alternative (AT) of the conveyor equipment. Please mark the text (VG, G, MG, M, MP, P and VP) based on the linguistic terms of experts' judgments. For example: Conveyor 1 has a very good speed. We mark "VG" in row "Speed" and column "AT1 for alternative 1".

Sub-criteria\Alternative (AT)	AT1	AT2	AT3	AT4
Convenient				
Maintainability				
Safety				
Risk				
Repeatability				
Purchasing cost				
Spare parts' cost				
Setting up… cost				
Speed				
Capacity				
…				
Flexibility				
…				
Easy…to use				
